# Objective Measurement of Ball-Handling Proficiency in Wheelchair Sports: A Systematic Review

**DOI:** 10.3389/fresc.2021.798675

**Published:** 2022-01-25

**Authors:** Viola C. Altmann, Barry S. Mason, Tijmen Geurts, Sanne A. J. H. van de Camp, Yves C. Vanlandewijck

**Affiliations:** ^1^Peter Harrison Centre for Disability Sport, Loughborough University, Loughborough, United Kingdom; ^2^Klimmendaal, Rehabilitation Centre, Arnhem, Netherlands; ^3^Department of Rehabilitation, Donders Institute for Brain, Cognition and Behaviour, Radboud University Medical Centre, Nijmegen, Netherlands; ^4^Department of Rehabilitation Sciences, Katholieke Universiteit (KU) Leuven, Leuven, Belgium; ^5^Swedish School of Sport and Health Sciences (GIH), Stockholm, Sweden

**Keywords:** wheelchair, ball handling performance, wheelchair sports, classification, sport specific activities, systematic review

## Abstract

**Background:**

In Paralympic sports, classification of athletes based on the impact of impairments on the ability to perform is needed, to prevent a one-sided and predictable outcome of the competition in which the least impaired athlete has the best chance to win. Classification is developing from expert opinion based to evidence based. In wheelchair court sports, there is evidence to support the impact of impairment on wheeled mobility, but not on ball handling. To assess the impact of impairment on the ability to perform ball-handling activities, standardised tests for ball handling are needed.

**Purpose:**

To assess if reliable and valid standardised tests for the measurement of ball-handling proficiency in a wheelchair or able-bodied court sports exist; to assist in the development of Evidence-Based Classification (EBC) in wheelchair court sports according to the guidelines of the International Paralympic Committee (IPC).

**Methods:**

The review was conducted according to the Meta-Analysis of Observational Studies in Epidemiology (MOOSE) statement. Search terms used were “wheelchair,” “ball,” “ball sports,” “test,” and “performance.” Databases searched were Medline, Embase, PubMed, and Sport Discus. Study quality was assessed using the Strengthening the Reporting of Observational Studies in Epidemiology checklist.

**Results:**

Twenty-two articles were included. Foundational Movement Skills in ball-handling proficiency were assessed. Tests for throwing maximal distance showed sufficient reliability and validity. Precision in throwing showed low-to-moderate reliability and conflicting results in validity. Throwing techniques differed between studies. Dribbling the ball showed high reliability, but conflicting results in validity.

**Conclusions:**

Tests for throwing maximal distance, throwing precision, and dribbling the ball can be used in standardised tests for activity limitation in wheelchair court sports. However, tests need to be adapted and standardised and then reassessed for reliability and validity in athletes with and without arm impairment.

## Introduction

The Paralympic Games are the third-largest sporting event in the world and provide an excellent platform to enhance participation and inclusion of persons with impairments in society (1). However, the value and the success of the Paralympic Games would become questionable, and the goal of participation would not be achieved if athletes who win the competition are simply the least impaired athletes. To prevent this, classification systems grouping athletes with impairments with a similar impact on performance in sports have been developed and applied since the start of the Paralympic Games (2). Typically, these classification systems were developed based on expert opinion by volunteer classifiers with a medical background and/or sport-specific expertise (3). The success of an athlete in competition can depend for a significant part on the class in which the athlete is competing. With the increasing professionalism of the Paralympic movement and the Paralympic athletes, a classification system based on expert opinion was no longer sufficient to support the value and success of the Paralympic Games, both for the athletes and society (4).

In 2007, the International Paralympic Committee (IPC) published the IPC Classification Code and International Standards to provide a structure for classification principles for all Paralympic Sports. In this Code, international sports federations were charged with the development of Evidence-Based Classification (EBC) systems through research (5). EBC means that the methods used to allocate sports classes must be based on scientific research, which demonstrates that the aim of classification, to group athletes for competition based on impairment severity with a similar impact on sport-specific performance, is achieved. The development of EBC systems requires four steps: (1) defining eligible impairment types per sport, (2a) developing valid and reliable measures of impairment, (2b) developing valid and reliable measures of determinants of sport-specific performance, and (3) assessing the relationship between impairment and performance determinants to define sports classes. Both the measures of impairment and performance determinants should be highly standardised, objective/instrumented, and ratio scaled where possible (6).

There are three wheelchair team court sports, wheelchair rugby (7), wheelchair basketball (8), and the newly developed sport wheelchair handball (9). The eligible impairment types for these three sports are neuromusculoskeletal impairments (strength, range of motion, coordination, and limb deficiency). Furthermore, these three sports have many commonalities in the activities that determine proficiency in the game. Based on the concept of Fundamental Motor Skills (10), these activities consist of locomotor skills, i.e., wheeled mobility, which is specific for wheelchair sports, and object control, i.e., ball handling, which shows much overlap with able-bodied sports. The term object control/ball handling is elaborated in the model of Foundational Movement Skills and consists of throwing with several techniques, bouncing/dribbling, and catching (11). Despite all commonalities, the classification systems of each of these sports, such as the eligibility criteria, the number of classes, and the criteria that define these sports classes, are completely different (12–14). Of the three classification systems, only the wheelchair rugby classification system is partially evidence-based (15). The evidence that is generated to support wheelchair rugby classification can potentially benefit the development towards EBC in wheelchair basketball and wheelchair handball. So far, wheelchair rugby classification is supported by evidence for trunk impairment in strength, range of movement and coordination (16, 17), and arm strength impairment (18). However, the relationship of these impairments with performance is only determined for wheeled mobility and not for ball handling (16–18). Therefore, in the interest of continuing the development towards EBC for all three wheelchair team court sports, step 2b develops valid and reliable measures of determinants of sport-specific performance, needs to be completed with tests for ball-handling proficiency. The definition we will use for ball handling in this study is based on the Foundational Movement Skills and consists of throwing with several techniques, bouncing/dribbling, and catching. These activities need specifications for wheelchair court sports. In wheelchair court sports, ball handling is restricted to handling a round ball with a size that is suitable for one- and two-handed direct manual ball handling without a device (like a bat or a stick). For throwing, both maximal distance and precision will be included as important aspects for proficiency.

The present study aimed to identify standardised tests for proficiency in ball handling according to the previously mentioned definition in team court sports (both Olympic and Paralympic) from the literature. The second aim was to assess if there is any evidence for the reliability and/or validity of these tests. The third aim was to determine if any or a combination of these tests can serve as a standardised test for ball-handling proficiency for the future development of EBC in wheelchair court sports.

## Materials and Methods

This systematic review was conducted and reported according to the consensus statement for the Meta-analysis Of Observational Studies in Epidemiology (MOOSE) (19), because based on the research question, the authors mainly expected to find observational studies. Two researchers performed the article search independently (TG and SC).

### Data Sources

Original articles were searched in Medline (1946–2020), Embase (1974–2020), PubMed (1989–2020), and Sport Discus (1949–2020). The following search terms were used for able-bodied sports: ball sports, performance, test, and arm or trunk. For wheelchair sports, wheelchair, ball, and performance were used as search terms. Search terms were linked with the Boolean AND. The search was extended using the option “related articles” in all databases. First, the title and abstract of the related articles were screened. If the title and the abstract met the inclusion criteria, the article was added to the numbers of identified records. In addition, the grey literature was also explored to ascertain whether any other articles outside the original search matched the criteria.

### Study Selection

Inclusion criteria for articles were (1) the outcome measures were ball handling with a round ball with a size that is suitable for one- and two-handed direct manual ball handling without a device and were presented in objective, quantitative data, (2) assessment for reliability was done, i.e., test-retest or inter-rater reliability and/or assessment of validity was done by the following comparisons: (a) between athletes playing a sport at different competition levels, (b) athletes with differences in age, (c) athletes with different physical characteristics of the arm or trunk (able-bodied sports), or (d) comparisons between participants with different levels of impairment (wheelchair sports), and (3) articles were written in English. Furthermore, for studies about able-bodied sports, (4a) participants were experienced athletes without impairments. Moreover, for wheelchair sports, (4b) the participants were experienced (sport) wheelchair users. To identify eligible articles, two reviewers independently screened the title and the abstract. If one reviewer found an article, both researchers screened for inclusion criteria. If the abstract met the criteria, then both researchers assessed the full text of the article. The article was assessed by a third researcher (VA) when there was a disagreement between the two reviewers. In this case, the third party had the final vote. If an article was found in more than one database, it was only included once.

### Quality Assessment

The methodological quality of each study was assessed independently by two reviewers (TG and SC) using the Strengthening The Reporting of Observational Studies in Epidemiology (STROBE) checklist for reports of observational studies (20). The STROBE checklist has 22 items, which were either scored as present (“1”) or absent (“0”). If one of the items had any sub-items, one point was awarded if the study met half or more of these sub-items. When disagreement existed on any item of the STROBE checklist, the same consensus procedure applied for inclusion criteria was used with three authors (TG, SC, and VA). The STROBE recommendations do not provide a guideline for including meaningful studies in a systematic review. However, in a study performed on the methodological quality of observational studies published in high-quality journals, an average of 69% of the STROBE items were reported (21). Consistent with this study and with others using quantitative cut-off scores for observational studies, we decided that a minimum of 15 reported items (69%) indicated “good quality,” whereas 14 reported items or less indicated “moderate-to-low quality.” Only studies with “good quality” were included in the discussion.

## Results

### Search Results

**Figure 1:**
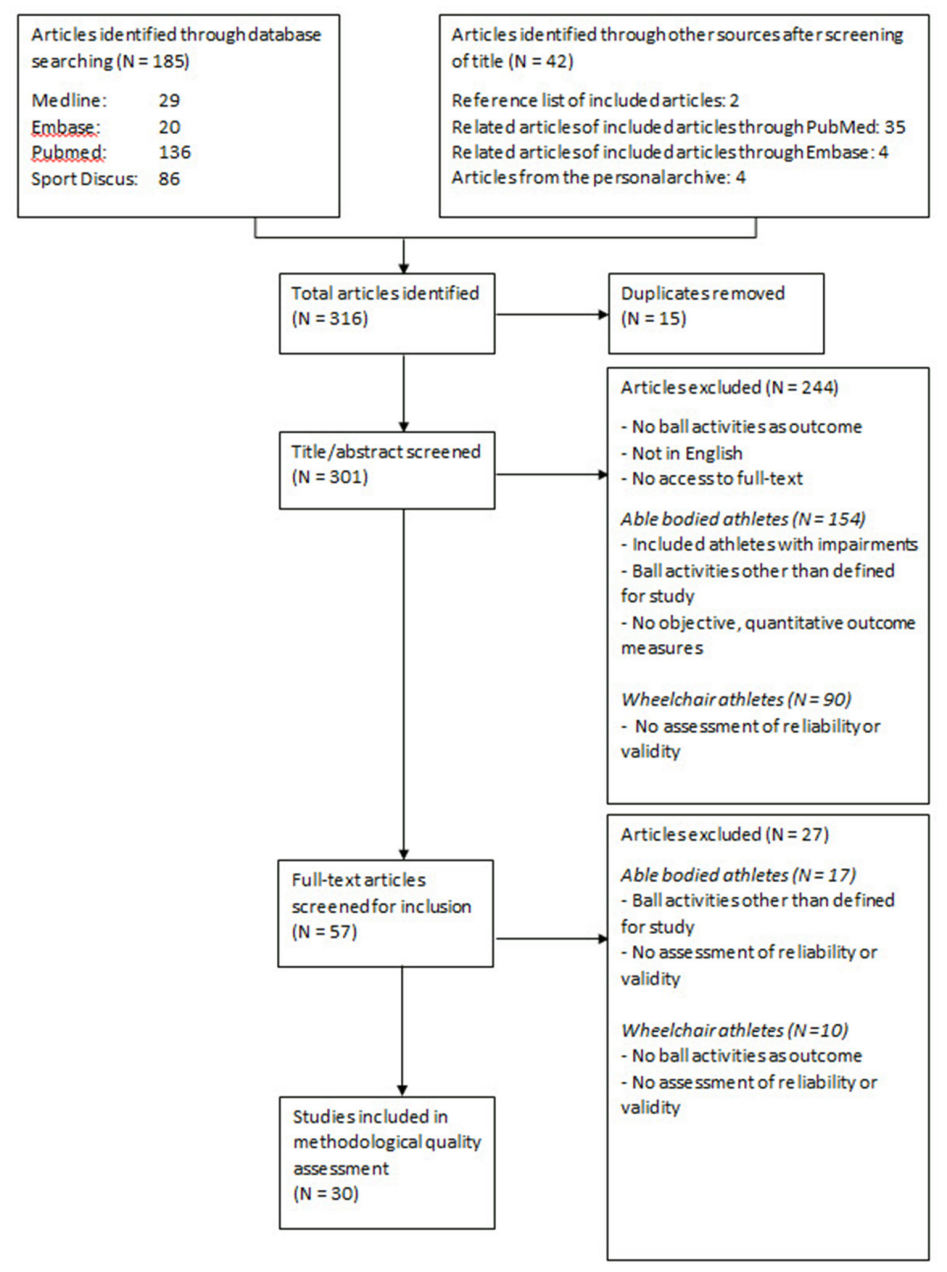
Flowchart of the literature search.

Figure 1 shows the number of articles found following each step of the search strategy. After database searching and searching additional sources, the researchers found 301 potentially relevant studies. After assessment for the inclusion criteria based on screening of titles and the abstracts, and if indicated, assessment of the full article, the researchers reached a consensus that 30 articles were eligible for methodological quality assessment. Most articles were excluded because the outcome measures were not based on ball handling as defined in the present study (handling a round ball with a size that is suitable for one- and two-handed direct manual ball handling without a device), or because there was no assessment of validity or reliability.

### Methodological Quality

The results of the quality assessment of the 30 articles that were eligible based on the inclusion criteria are shown in Table 1.

**Table 1:** Study participants, interventions, comparisons, and STROBE scores.

**References**	**Participants**	**Intervention**	**Comparison**	**Total score STROBE**	**Methodological quality**
Barfield et al. (22)	*WC rugby* 10 WRP national team and 10 WRP not national team	Long pass Short pass Slalom with ball	WRP national team, WRP not national team	13	Moderate—poor
Bayios et al. (23)	*AB handball* 15 FD, 12 SD male HP and 15 PE students	Throwing on the spot and with cross-over step and vertical jump shot	FD, SD, PE students	15	High
Bayios et al. (24)	*AB handball* 15 FD, 12 SD, 15 PE students	Ball throw on the spot and with cross-over step	FD, SD, PE students	13	Moderate—poor
Borges et al. (25)	*WC handball* 21 WHP Low point (1.0–1.5) 7 athletes Midpoint (2.0–2.5) 6 athletes High point (3.0–4.0) 9 athletes	Slalom with ball	Low, mid and high point WHP	14	Moderate—poor
Cavedon et al. (26)	*WC basketball* Class A (0.5) 18 athletes Class B (1.0–1.5) 16 athletes Class C (2.0–2.5) 8 athletes Class D (3.0–4.0) 9 athletes	Maximal pass, pass for accuracy, spot shot, lay-ups, 20 m sprint with ball	FAC 1 vs. 2 vs. 3 vs. 4	19	High
Cerrah (27)	*AB soccer* 14 male soccer players	Throwing the ball in while standing and running	Isokinetic strength of the arms (shoulder and elbow) and the trunk flexion and extension	12	Moderate- poor
Costa e Silva (28)	*WC handball* 29 WHP Group 1 (1.0–1.5) 6 players Group 2 (2.0–2.5) 8 players Group 3 (3.0–3.5) 6 players Group 4 (4.0–5.0) 9	Throwing against the wall and catching Slalom with the ball	Group 1, 2, 3 and 4	10	Moderate- poor
Debanne et al. (29)	*AB handball* 12 high national, 17 high regional and 13 local male HP	Standing overarm throw	No group comparisons	17	High
Erculj et al. (30)	*AB basketball* 23 division A European players 25 division B European players	Basketball throw Medicine ball (2 kg) throw	Division A vs. division B players	8	Moderate- poor
Fieseler et al. (31)	*AB handball* 12 FD, 34 TD male HP	Throws with run-up or jump overarm throw with and without precision	FD vs. TD	20	High
Garcia-Gil et al. (32)	*AB basketball* 41 FD female BP from 4 teams in first division national league Spain with varying placements	Dribbling test	4 FD teams with varying placements	17	High
Gil et al. (33)	*WC basketball* 13 WBP Class 1.0, 1 athlete; class 1.5 1 athlete; class 2.0 3 athletes; class 2.5 1 athlete; class 3.0 2 athletes; class 3.5 2 athletes; class 4.0 2 athletes; class 4.5 1 athlete	Pick-up the ball, maximal pass with basketball and medicine ball (5 kg), 20 m sprint with the ball	Athlete class and injury type (SCI or non-SCI), years of experience in WC and years of experience in WC basketball	19	High
Gorostiaga et al. (34)	*AB handball* 15 FD and 15 SD male HP	Standing throw and 3-step running throw	FD vs. SD And correlation with arm strength and power production	18	High
Granados et al. (35)	*AB handball* 16 FD and 15 SD female HP	Standing throw and 3-step running throw	FD vs. SD	18	High
Granados et al. (36)	*AB handball* 16 national and 14 international female HP	Standing throw and 3-step running throw	National vs. international	20	High
Granados et al. (37)	*WC basketball* 19 FD and TD WBP	Anthropometric and performance values. Ball pick-up, maximal pass with	FD vs. TD	18	High
		two arm overhand with basketball and medicine ball (5 kg), 20-m sprint with ball including dribble			
De Groot et al. (38)	*WC basketball* 19 WBP Class 1.5, 2 athletes Class 2.0, 2 athletes Class 2.5, 3 athletes Class 3.5, 1 athlete Class 4.0, 5 athletes Class 4.5, 6 athletes	Pass for accuracy, free-throw shooting, 20 m sprint with the ball, maximal pass, lay-ups, pick-up the ball, spot shot	Premier league vs. tournament A vs. tournament B. Trial 1 vs. 2.	18	High
Marszalek et al. (39)	*WC basketball* 29 class A (1.0–2.5), 29 athletes class B (3.0–4.5) 32 athletes	Basketball chest pass test	Class A vs. class B	16	High
Marszalek et al. (40)	*WC basketball* 9 WBP	Two handed pass basketball and medicine ball (3 kg)	The first vs. second repetition of the tests	16	High
Molik et al. (41)	*WC basketball* 109 WBP Class 1, 26 athletes Class 2, 25 athletes Class 3, 24 athletes Class 4, 16 athletes Class 4.5, 18 athletes	Two handed chest pass Slalom with the ball	Differences between athlete classes	12	Moderate-poor
Moss et al. (42)	*AB Handball* 47 non-elite, 44 elite and 29 top-elite female youth HP	Standing throw and 3-step running throw	Top-elite, elite and non-elite	17	High
Ortega et al. (43)	*AB Handball* 13 elite, 16 U18 and 16 U16 male HP	3 step running throw and jump throw	Elite, U18 and U16	18	High
Saavedra et al. (44)	*AB Handball* 23 A-team, 16 U19, 20 U17 and 21 U15 national team female HP	Standing throw	A-team, U19, U17, U15	16	High
Schwesig et al. (45)	*AB Handball* 30 male TD HP	Bal throwing with cross-step and throwing time	No group comparisons	18	High
Tachibana et al. (46)	*WC basketball* Class 1 (1.0–1.5) 7 athletes Class 2 (2.0–2.5) 7 athletes Class 3 (3.0–3.5) 5 athletes Class 4 (4.0–4.5) 8 athletes	Figure of eight with ball, pass for distance in chest-pass, baseball-pass and hook-pass	Wheelchair basketball class	18	High
Visnapuu et al. (47)	*AB Handball* 34 10–11 year, 39 12–13 year, 39 14–15 year and 21 16–17 year old male HP	30 m dribble test, handball throw from sitting position, passing on speed and precision	10–11, 12–13, 14–15 and 16–17 years old	14	Moderate—poor
Wagner et al. (48)	*AB Handball* 5 FD, 12 FoD and SiD male HP	Game based performance test including catching and passing ball as fast as possible	2 tests separated by 7 days	18	High
Yanci et al. (49)	*WC basketball* 14 males, 2 females Category A (class 1.0–2.5) 7 athletes Category B (class 3.0–4.5) 9 athletes	Pick-up the ball, maximal pass, 5 and 20 m sprint with ball	FAC A (1.0 to 2.5) vs. FAC B (3.0 to 4.5)	16	High
Yilla et al. (50)	*WC rugby* 65 WRP with quadriplegia. 60 had spinal cord injuries, 2 poliomyelitis, 1 muscular dystrophy, 1 charcot-marie-tooth syndrome, 1 cerebral palsy	Pass for accuracy, catching, pass for distance	The first vs. second repetition of the tests Players rank, determined by experts	18	High
Yüksel et al. (51)	*WC basketball* 12 FD, 9 SD WBP	Pass for distance, lay-up tests, zone shot test, slalom with ball, pass for accuracy test	FD vs. SD	16	High

*STROBE, strengthening the reporting of observational studies in epidemiology; WC, wheelchair; AB, able bodied; FD, first division; SD, second division; TD, third division; FoD, fourth division; SiD, sixth division; HP, handball players; BP, basketball players; WBP, wheelchair basketball players; WRP, wheelchair rugby players; WHP, wheelchair handball players; FAC, functional ability class; U, under (age limit); PE, Physical Education. Articles with a total STROBE score of ≥ 15 were included in the study*.

### Findings of the Review

Twenty-two articles fulfilled the predetermined minimum of 15 reported items on the STROBE checklist (see Table 1) (22–51). They all had a cross-sectional design. Twelve high-quality studies were about able-bodied sports, 11 about handball (23, 29, 31, 34–36, 42–48), and 1 about basketball (32). Two articles (23, 24) were about the same study, of which only one was “high quality.” 10 high-quality studies were about wheelchair sports, 9 about wheelchair basketball, (26, 35, 37–40, 46, 49, 51) and 1 about wheelchair rugby (49). Wheelchair athletes had health conditions, such as spinal cord injury, spina bifida, cerebral palsy, neuromuscular conditions, and congenital and acquired amputations, leading to all eligible impairment types for wheelchair rugby, wheelchair basketball, and wheelchair handball (strength impairment, coordination impairment, impaired range of movement, and limb deficiency**)**.

In all but one study, throwing for accuracy and distance using different throwing techniques was assessed. The most frequently used outcome parameters for throwing were ball velocity (12 tests) and throwing distance (13 tests). For accuracy, 12 studies used the number of scores on the target; the target was usually a basketball bucket. Only one study measured throwing accuracy in continuous data, the surplus in centimetres by which a projected target was missed, but this study was rated as moderate-poor quality (24). In 10 studies, (26, 32, 33, 37, 38, 41, 46, 47, 49, 51) running or pushing with the ball, such as the dribbling rules of the game, was assessed. In addition, in four of these studies, picking up the ball from the floor during wheelchair pushing was assessed (33, 37, 38, 49). All these tests involved running or wheeling, and the time for completion of the test was used as the outcome parameter. In only one study catching was included (50).

Test-retest reliability was included in seven studies (37, 38, 40, 43, 45, 48, 50). For outcomes of these studies, see Table 2.

**Table 2:** Reliability.

**Ball proficiency item**	**Outcome parameter**	**Reliability**	Study numbers^*^
Throwing maximal distance	Throwing velocity (m/s) or distance (m)	High	(**37**, **38**, **40**, 43, 45, 48,**50**) (**37**) (medicine ball 5 kg) (**40**) (medicine ball 3 kg)
Throwing accuracy	Score in basket or goal (n)	Moderate-low	(**38**,^45^, ^48^)
Ball handling while pushing	Time to complete trajectory (s)	High	(**37**, **38**, **50**)
Catching the ball	Balls caught (n)	High	(**50**)

* *Study numbers with participants without impairments/running sports.*

***Study numbers with participants with impairments/wheelchair sports***.

Test-retest reliability was high for all tested items of ball proficiency, except for throwing accuracy.

All but one study included a measure for validity. For outcomes of these studies, see Table 3.

**Table 3:** Validity.

**Ball proficiency item**	**Comparison**	**Outcome parameter**	**Difference between groups (yes/no/conflicting)**	Study numbers^*^
Throwing maximal distance	Competition divisions	Throwing velocity (m/s)	Yes	(23, 31, 32, 42, 44)
	Competition divisions	Throwing distance (m/s)	Yes	(**26**, **37**, **38**) (**37**) (medicine ball 5 kg)
	Strength or anthropometric data	Throwing velocity (m/s)	Yes	(23, 29, 31, 34–36, 42–44)
	National- international athletes	Throwing distance (m)	No	(36)
	Impairment classes	Throwing distance (m)	Conflicting	(**26**, **33**, **39**, **46**, **49**) (yes) (**38**) (no)
Throwing accuracy	Divisions	Score in basket or goal (n)	Yes	(**38**, **51**)
	Impairment classes	Score in basket or goal (n)	Conflicting	(**26**) (yes) (**38**) (no)
	Player ranking by experts	Score in goal	No	(**38**, **50**)
Ball handling while pushing or running	Divisions	Time to complete trajectory (s)	Conflicting	(**38**) (yes) (32,**37**, **49**) (no)
	Impairment classes	Player ranking by experts	Conflicting	(**33**) (only difference for athletes with spinal cord injury, but not for athletes with other health conditions) (**26**) (no)
	Player ranking by experts	Player ranking by experts	Yes	(**50**)
Catching the ball		Balls caught (n)	No	(**50**) (ceiling effect, in which 90% of all athletes achieved maximum score)

* *Study numbers with participants without impairments/running sports.*

***Study numbers with participants with impairments/wheelchair sports***.

The groups that were compared were either based on performance (competition divisions, player ranking by experts, or national vs. international players) or on impairment classes. For maximal throwing distance, there was a difference between groups in almost all studies. The only exceptions were national vs. international athletes in one study and impairment classes in one out of five studies. No differences between groups were found in many studies for throwing accuracy and ball handling while running or pushing. There were no differences between groups in one study for catching the ball due to an important ceiling effect.

## Discussion

### Summary of the Evidence

In this systematic review, we synthesised the evidence on the reliability and validity of standardised tests for ball-handling proficiency in court sports, available in the literature. As anticipated, all studies identified were observational studies. In the studies about wheelchair sports, all impairment types that are eligible for wheelchair rugby, wheelchair basketball, and wheelchair handball were included. The evidence indicated that tests for maximal throwing were both reliable (37, 38, 40, 43, 45, 48, 50) and valid (23, 26, 29, 31, 33–37, 39, 42–44, 46, 49) in relation to competition level, anthropometric data, and impairment classes. Tests for throwing accuracy lacked reliability (38, 45, 48), and validity showed conflicting results (26, 38, 50, 51). Besides, the correlation with player ranking as a measure for game performance in wheelchair rugby was low (50). Dribbling or bouncing the ball during running or wheeling showed high reliability (37, 38, 50), but validity showed conflicting results (26, 32, 33, 37, 38, 49, 50). Finally, there was only one study in which catching was included (50). This test showed high reliability but the validity was limited by a large ceiling effect in wheelchair rugby athletes.

Tests for maximal throwing distance showed both adequate reliability and validity for potential use as a measure for sport-specific ball-handling proficiency that can be used in the development for EBC (23, 26, 29, 31, 33–40, 42–46, 48–50). The outcome measure can be distance, (26, 33, 39, 46, 49) which can be measured with limited equipment. However, this test requires a rather large testing area as distances of more than 15 m can be thrown (46). Limitations of room size can be addressed by using a medicine ball (3–5 kg) instead of a normal ball, which reduces the maximal distance to ~5 m. (37). However, athletes with severe arm and trunk impairment may not be able to throw such a heavy ball. Another good alternative is using throwing velocity as the outcome measure (23, 29, 31, 34–36, 42–44). However, this requires equipment, such as like laser beam emitters and laser beam infrared detectors, (23) a Doppler-radar gun (29, 42, 44), a speed check radar device, (31, 43, 45) or photocells (34–36). However, the objectivity and precision of measuring velocity may be superior to measuring distance, as the distance was measured with a tape measure where the ball was observed to touch the floor instead of with instrumented equipment.

In studies about able-bodied sports, the throwing technique was an overarm, one-handed pass with the dominant arm in either a standing throw, three-step running throw, cross-over-step throw, or a jump throw (23, 29, 31, 34–36, 42–45). In studies about wheelchair sports, all tests were performed standing still, and several throwing techniques were used. Most studies included a chest pass (39, 40, 46) or two-arm overhand pass (37). In several studies, the technique was not specified (26, 33, 38, 49, 50). In only one study, one-handed passes (baseball and hook pass) were assessed (46). Because arm and hand impairment, which is present in all wheelchair rugby players and in part of the wheelchair basketball and wheelchair handball players, can impact the throwing technique, the throwing technique should be standardised, and preferably, both two-handed and one-handed techniques should be included.

Based on the findings in the literature, we advise including maximal throws in the standardised test of ball-handling proficiency for testing of sport-specific performance in wheelchair court sports. Preferably, maximal throws should include standardised techniques for two-handed and one-handed throws. Outcome measurement in ball velocity has advantages over distance in measurement precision and the room needed for the tests.

Throwing distance in court sports is meaningless if the throw does not reach a target. Throwing accuracy is important for successfully passing the ball to other players resulting in a catch in all three wheelchair court sports and for scoring a goal in wheelchair basketball and wheelchair handball (7–9). Throwing accuracy was assessed in several studies (26, 38, 45, 48, 50, 51). In three studies, the number of scores in the bucket was used as an outcome parameter (26, 38, 51), and in two scores in a handball goal (45, 48). Scoring or not scoring is a binary parameter and the difference between scoring and not scoring can be minimal. In only one study, circles around a goal with a maximum score in the middle circle and decreasing scores in the outer circles were used (50). However, this still results in a score on an ordinal scale, where a ratio scale is advised (6). In one study, two-handed throws were used (51), and in two studies, one-handed throws were used (45, 48). In the other studies, the throwing technique was not specified (26, 38, 50). The reliability for throwing accuracy was low in all studies in which this was assessed (38, 45, 48). Perhaps this is due to the binary or ordinal scale that was used, in which a difference of several millimetres in a throw of several metres can make the difference between a score and no score or scoring points. If an interval scale would be used, measuring the surplus from the goal in centimetres of millimetres, a variation of several centimetres or millimetres between throws will result in less difference between measures than “hit” or “no hit,” and reliability would increase. Furthermore, scoring as an outcome measure for throwing accuracy, using only one throwing technique is rather limited in comparison to the repeated throws between players and multiple throwing techniques that can be used in a game (7–9). It is striking that the only study in which catching was assessed showed a large ceiling effect (50). Accuracy of a throw plays an important role in catching the ball. However, in the study, the precision of the throw after which the ball needed to be caught was not specified. Maybe the high precision of the throw explains the ceiling effect that was found in this test. A more game-specific measure of throwing accuracy may be throwing at a target and the outcome measure is the surplus (proximity to the target in cm), measured in an interval scale. The latter was done in one study (24). However, this research article had moderate-to-low quality and the measurement device, and the methods were not described clearly enough to be repeated. Based on the findings in the literature, methods for throwing accuracy need to be developed and reliability and validity need to be assessed. Throwing at a target using surplus as the outcome parameter in an interval scale seems an interesting option to test throwing accuracy. Similar to tests for throwing distance, tests for throwing accuracy preferably should include standardised techniques for two-handed and one-handed throws. In addition, there may be a relationship between throwing distance and throwing accuracy, in which accuracy decreases if the throwing distance increases to the maximum throwing distance. However, this was not assessed in any of the studies. Tests for throwing accuracy using different percentages of the maximal throwing distance may reveal such a relationship, which is very important for proficiency in ball handling. Furthermore, we advise including tests for catching after standardised throws with more or less velocity and precision, such as can be done by a ball launcher.

Finally, dribbling or bouncing the ball within the game rules, while moving the wheelchair is an activity that contributes to proficiency in wheelchair court sports. Dribbling the ball while running or moving the wheelchair was assessed in several studies (26, 32, 33, 37, 38, 49, 50), of which the ones about wheelchair basketball (26, 33, 37, 38, 49) and wheelchair rugby (50) will be most specific for wheelchair court sports. Reliability was high for picking up the ball (37, 38), 20 m sprint with the ball (37), and manoeuvrability with the ball (50). Assessment for validity showed promising results in several studies about wheelchair sports. Differences were found between impairment classes, but only for athletes with spinal cord injuries (33), and between divisions (38) and player ranking (50). However, in four studies, no differences were found between impairment classes (26, 38, 49) and between divisions (37). Impairment classes in wheelchair basketball are defined by trunk active range of movement (13), and it is known from previous studies that the velocity in pushing a wheelchair largely depends on impairment in arm muscle strength (16, 18). Because the outcome measure was time to complete the test and pushing made up a large part of the time to complete the test, this may have obscured any differences between impairment classes in ball handling during pushing. Performing two tests on the same circuit, one with and one without dribbling and bouncing the ball and then subtracting the results of these tests could minimise the impact of pushing the chair and give more insight into the component of dribbling and bouncing the ball (52).

If athletes with different severities of arm and hand impairment will be included, it is likely that differences in test performance will be found, which will support the validity of tests for dribbling and bounding the ball.

Based on these findings, we advise including standardised tests for dribbling or bouncing the ball while moving the wheelchair in a test battery for ball-handling proficiency. The same circuit should be done with and without the ball handling and the times to complete the test should be subtracted to eliminate the impact of pushing on the outcome from the test. Athletes can push in a straight line or in a circuit and ball handling should include picking up the ball and dribbling. Validity in relation to impairment severity needs additional assessment including athletes with arm impairment.

### Strengths and Limitations

This systematic review has several strengths. First, the strict study protocol using the MOOSE standard for meta-analysis (19) enables replicating the study and extending it in the future if new evidence will become available. Second, several types of bias were considered and minimised. Publication bias was minimised by extending the search to the grey literature. Both bias in selection for study inclusion and bias in the methodological quality assessment were addressed, respectively, by independent literature searches and independent assessment of study quality by two researchers (SC and TG). Finally, bias in results and conclusions was minimised by using the STROBE guideline for methodological assessment of observational studies (20).

There are several limitations that need to be considered when interpreting the results of this literature review. First of all, the studies included had rather small study populations, ranging from 14 to 120 participants in able-bodied sports (27, 42), and 13 to 65 participants in wheelchair sports, (33, 50) which limited the power of each of the studies. This may have obscured differences between groups for the assessment of validity, especially in activities with limited reliability, such as throwing precision. Pooling of the data from several studies to increase the power was not possible, because study populations, throwing techniques, measurement techniques, and outcome measures were different across the studies.

There may have been biases within the studies that were included, based on several issues. In most studies about wheelchair sport, the relationship between the activity and the wheelchair basketball classification was used as a measure for severity of impairment (26, 33, 38, 39, 46, 49). However, these wheelchair basketball classes are not an evidence-based measure of impairment severity (11, 13). Furthermore, several wheelchair basketball classes were grouped for analysis, to increase the number of athletes per group (26, 39, 46, 49). This may have increased the variation of impairment severity within groups, which may have obscured any difference between groups even more. In several studies, the technique for throwing, dribbling, and picking up the ball was not specified (26, 33, 37, 38, 49, 50), and therefore may not have been the same for all participants. This may have limited the variation in the outcome measures for throwing velocity and precision between groups because participants could compensate for their limitations by altering the throwing technique. Furthermore, it was not always clearly described if athletes were allowed to use equipment, such as sticky material on the hands or the ball in handball and gloves in wheelchair rugby. This may also have limited the differences between groups for both throwing velocity and precision. Last but not the least, only studies with experienced athletes were included in this review. However, the levels of experience and training were different across the studies and ranged from recreational athletes training only once a week (26) to elite international athletes competing at the highest level (43). This may have caused considerable variation within groups, obscuring differences between groups. All these forms of bias may have affected the conclusions about the reliability and the validity of the studies. For the development of a standardised test battery for ball-handling proficiency, it will be important to recruit enough optimally trained athletes to participate for sufficient study power. This will be a challenge, because the number of elite wheelchair athletes is limited, and they are spread geographically. However, because aspects of ball-handling proficiency are similar for the three wheelchair team court sports, combining athletes from these sports may help overcome this obstacle. Furthermore, ball activities should be standardised, such as throwing, ball pick-up and dribbling techniques, and the use of equipment.

## Conclusions

The findings of this review provide valuable information for the development of a standardised test battery for ball activities in team wheelchair court sports. Based on the findings, we advise a test battery, which includes at least all Foundational Movement Skills for ball handling, throwing with several techniques, bouncing/dribbling, and catching. Throwing should include standardised throwing techniques with at least a two-handed and a one-handed throw. Throwing should be assessed for maximal distance and accuracy including the relationship between distance and accuracy. Bouncing/dribbling the ball should include a standardised pushing distance and trajectory, i.e., picking up and dribbling the ball. This activity can be assessed using execution time if the impact of pushing on the test is minimised. Assessment of the test-retest reliability and the validity of the test battery needs to be assessed before this test battery can be used in the steps in the development of EBC that follow steps 1) define eligible impairment types and 2a) develop valid and reliable measures of impairment. These steps are 2b) developing valid and reliable measures of sport-specific performance, and 3) assessing the relationship between impairment and performance to define sports classes.

## Data Availability Statement

The original contributions presented in the study are included in the article/supplementary material, further inquiries can be directed to the corresponding author/s.

## Author Contributions

VA, BM, and YV formulated the research question and they established the study design. VA, BM, TG, SC, and YV contributed to the manuscript, discussed the study results, and the relevance with regards to the research questions. TG, SC, and VA performed the check for inclusion criteria and the assessment of the quality of the selected studies. TG and SC performed the literature search. All authors contributed to the article and approved the submitted version.

## Funding

The publication fee will be paid/refunded by World Wheelchair Rugby https://worldwheelchair.rugby/ Contact person Steve Griffiths, CEO and secretary general.

## Conflict of Interest

The authors declare that the research was conducted in the absence of any commercial or financial relationships that could be construed as a potential conflict of interest.

## Publisher's Note

All claims expressed in this article are solely those of the authors and do not necessarily represent those of their affiliated organizations, or those of the publisher, the editors and the reviewers. Any product that may be evaluated in this article, or claim that may be made by its manufacturer, is not guaranteed or endorsed by the publisher.
